# Immune and Inflammatory Cell Composition of Human Lung Cancer Stroma

**DOI:** 10.1371/journal.pone.0139073

**Published:** 2015-09-28

**Authors:** G-Andre Banat, Aleksandra Tretyn, Soni Savai Pullamsetti, Jochen Wilhelm, Andreas Weigert, Catherine Olesch, Katharina Ebel, Thorsten Stiewe, Friedrich Grimminger, Werner Seeger, Ludger Fink, Rajkumar Savai

**Affiliations:** 1 Internal Medicine, University of Giessen and Marburg Lung Center, Member of the German Center for Lung Research, Giessen, Germany; 2 Department of Lung Development and Remodeling, Max-Planck-Institute for Heart and Lung Research, Member of the German Center for Lung Research, Bad Nauheim, Germany; 3 Institute of Biochemistry I, Goethe-University Frankfurt, Frankfurt, Germany; 4 Molecular Oncology, Philipps-University, Member of the German Center for Lung Research, Marburg, Germany; 5 Institute of Pathology and Cytology, UEGP, Wetzlar, Germany; Universitatsklinikum Freiburg, GERMANY

## Abstract

Recent studies indicate that the abnormal microenvironment of tumors may play a critical role in carcinogenesis, including lung cancer. We comprehensively assessed the number of stromal cells, especially immune/inflammatory cells, in lung cancer and evaluated their infiltration in cancers of different stages, types and metastatic characteristics potential. Immunohistochemical analysis of lung cancer tissue arrays containing normal and lung cancer sections was performed. This analysis was combined with cyto-/histomorphological assessment and quantification of cells to classify/subclassify tumors accurately and to perform a high throughput analysis of stromal cell composition in different types of lung cancer. In human lung cancer sections we observed a significant elevation/infiltration of total-T lymphocytes (CD3^+^), cytotoxic-T cells (CD8^+^), T-helper cells (CD4^+^), B cells (CD20^+^), macrophages (CD68^+^), mast cells (CD117^+^), mononuclear cells (CD11c^+^), plasma cells, activated-T cells (MUM1^+^), B cells, myeloid cells (PD1^+^) and neutrophilic granulocytes (myeloperoxidase^+^) compared with healthy donor specimens. We observed all of these immune cell markers in different types of lung cancers including squamous cell carcinoma, adenocarcinoma, adenosquamous cell carcinoma, small cell carcinoma, papillary adenocarcinoma, metastatic adenocarcinoma, and bronchioloalveolar carcinoma. The numbers of all tumor-associated immune cells (except MUM1^+^ cells) in stage III cancer specimens was significantly greater than those in stage I samples. We observed substantial stage-dependent immune cell infiltration in human lung tumors suggesting that the tumor microenvironment plays a critical role during lung carcinogenesis. Strategies for therapeutic interference with lung cancer microenvironment should consider the complexity of its immune cell composition.

## Introduction

Lung cancer is a highly aggressive and challenging disease and is the leading cause of cancer mortality worldwide. Despite ongoing therapeutic efforts, lung cancer patients have a poor prognosis with an average 5-year survival rate of only 15% [1] [2]. Approximately 80–85% of all lung cancer patients are treated with one or more options within a standard regimen that involves surgery, radiation therapy, and chemotherapy with disease stage determining the therapeutic options. Although these treatments have produced promising results as neo-adjuvant and adjuvant strategies for early-stage patients and for treatment of locally advanced and advanced disease, treatment outcomes for lung cancer are still considered disappointing. This is largely due to a delay in diagnosis and inadequate knowledge about tumor progression and its associated molecular alterations [3]. Important advances have recently been made in identifying the molecular determinants of carcinogenesis, such as genetic alterations in many oncogenes (*Kras*, *cMyc*, *EGFR*, *ALK*, etc.) and tumor-suppressor genes (*p53*, *RASSF1*, *RB*, *FHIT*) [4, 5].

In addition to this genetic complexity, the cellular complexity of the tumor microenvironment is increasingly recognized as contributing directly to cancer initiation, progression and metastasis [6, 7]. The tumor microenvironment, depending on the tumor location, is composed of stromal cells including fibroblasts, immune and inflammatory cells, adipocytes, glial cells, smooth muscle cells and resident and recruited vascular cells along with the extracellular matrix, growth factors/cytokines and other proteins that are locally and/or systemically produced. Although none of these stromal cells are tumorigenic, they may either stimulate or inhibit cancer cell proliferation/malignancy depending on the tumor microenvironment and the various interactions they may have with the cancer cells [8, 9].

Although immune cells should in principle detect and eliminate transformed cells, their interaction with tumor cells may lead to changes in their phenotype that may actually result in the establishment of a tumor-supporting environment in various cancer settings, including lung cancer [10–12]. Thus, a comprehensive analysis of the population/ composition of stromal cells and a better understanding of their impact on the process of carcinogenesis may eventually lead to improved anticancer therapies [13, 14]. Along this line, there is now growing evidence that certain immune cells infiltrate into the tumors of human samples of lung cancer [12, 15–19]. However, to the best of our knowledge, the identification and quantification of several immune cell populations and their correlation to lung cancer type, stage and nodal status has not been reported. In this study, employing tissue arrays and immunohistochemistry, we substantially extended this characterization to include several immune cell populations as well as different lung cancer types, cancer stages, and tumor sizes as well as differences in nodal status. These techniques were combined with cyto-/histomorphological assessment and quantification of the cells, to classify/subclassify tumors accurately and high throughput analysis of stromal cell composition in different types of lung cancer.

## Materials and Methods

### Lung Specimens

Lung cancer tissue array, LUC1501 contains 150 cores from normal/benign (3 cases) and cancer (70 cases with grading and TNM staging data), duplicated cores per case were purchased from Pantomics, Inc. (Cat no. LUC 1501; Richmond, CA, USA). All the tissues were fixed in 10% neutral buffered formalin for 24 hours and processed using identical SOPs. Sections were picked onto Superfrost Plus or Startfrost adhesive slides. There may be >5% core loss per slide but the core retention rate should be >90%. The tumor specimens were presented in duplicates for internal control and to assess tumor heterogeneity. In addition, a pathologist validated the tumors in the cores. The tumors cover between 50 and 100% of the cores. Six additional samples of donor lung tissue were taken from lungs that were not transplanted [20]. This donor lung tissue was non-transplanted lung tissue of transplant donors. The study protocol for tissue donation was approved by the ethics committee (“Ethik Kommission am Fachbereich Humanmedizin der Justus Liebig Universität Giessen”) of the University Hospital Giessen (Giessen, Germany) in accordance with national law and with “Good Clinical Practice/International Conference on Harmonisation” guidelines. Written informed consent was obtained from each patient or the patient’s next of kin (AZ 31/93) [20, 21]. All specimens were analyzed under a Hamamatsu NDP slide scanner (Hamamatsu Nanozoomer 2.0HT) and its viewing platform (NDP.Viewer).

### Hematoxylin and Eosin Staining

Lung cancer tissue array was deparaffinized in xylene followed by rehydration in 100%, 90%, and 70% ethanol and distilled water. The slides were then incubated in fresh hematoxylin (Merck, Darmstadt, Germany) for 20 min and washed in distilled water, followed by incubation in acidified eosin solution (Sigma, Deisenhofen, Germany) for 1 min and washing. Finally, the slides were dehydrated in 90% and 100% ethanol, air dried, and mounted [20].

### Immunohistochemistry

Immunohistochemical staining was performed using a Autostainer Plus (Dako, Hamburg, Germany) and mouse monoclonal antibodies from Dako, Medac (Hamburg, Germany), and Thermo Fisher (Dreieich, Germany) at dilutions shown in Table 1. A polyclonal antibody was only used for myeloperoxidase (MPO) staining. We followed the specific standardized protocol supplied by the manufacturer. Omission of the primary antibody served as a negative control. Briefly, slides were pretreated with Trilogy buffer (Medac; 1:100, 16 min at 95°C), citrate low buffer (Thermo Fisher; 1:100, 26 min at 98°C), or pronase E (Merck; 0.1%, 10 min at room temperature) followed by treatment with 3% H_2_O_2_ for 8 min. All antibodies were applied in a volume of 200 μl and incubated for 30 min. After washing (Medac wash buffer, 1:20 in aqua dest), secondary antibody (Medac) was applied in a volume of 200 μl and incubated for 20 min. After washing, each sample was incubated with polymer (200 μl) for 30 min (note that the secondary antibody and polymer are components of the color-coded BrightVision HRP kit from Medac). The slides were washed twice and incubated in Bright DAB (Medac) for 10 min. The slides were washed in aqua dest, counterstained with hematoxylin for 8 min, and washed before coverslipping [20, 22].

**Table 1:** Antibody details.

Epitope	Clone	Dilution	Pretreatment	Supplier
CD3	F.2.38	1:400	Trilogy	Dako
CD4	4B12	1:1	Trilogy	Thermo Fisher
CD8	C8/144B	1:100	Trilogy	Medac
CD20	L26	1:1000	Citrate low	Dako
CD117/c-kit	C-kit	1:200	Citrate low	Medac
CD68	PGM-1	1:1000	Citrate low	Medac
CD11c	5D11	1:100	Trilogy	Dako
MUM-1	Mum1p	1:100	Trilogy	Dako
PD-1	MRQ22	1:100	Trilogy	Medac
Myeloperoxidase	Polyclonal	1:2000	Pronase E	Dako

### Data Analysis and Statistics

The total number of cells and positively stained cells were counted in tissue sections. In combination with an immunohistochemical stain, we also relied on a cyto-/histomorphological assessment of the cells by a pathologist. The H&E stained TMA sections were assessed by pathologist for differentiating the tumor part from the non-tumor part The total number of cells and all the positively stained cells in tumor area of the core were counted. The values represent the cell count for each cell-type marker per 1000 total cells counted. The differences in these normalized counts between groups were estimated with a generalized linear model of the quasi-Poisson family with log-link. This model assumes that the response values follow a Poisson distribution, as is expected for counts. Analysis of the residuals indicated an overdispersion, that is, there was not a linear relationship between the mean and variance, a characteristic of purely Poisson-distributed data. This was accounted for by quasi-likelihood estimation of the Poisson model including an additional scale parameter [23]. Presented data are the predicted responses for the groups (means or expected values) with 95% confidence intervals (CI). P-values for the comparison of groups were also calculated for these models. Data from TNM classifications T2 and T3 were pooled for analysis. Data were analyzed with R version 3.1.0 [24, 25].

## Results

### Histopathological Analysis of Lung Tumor Samples

**Fig 1:**
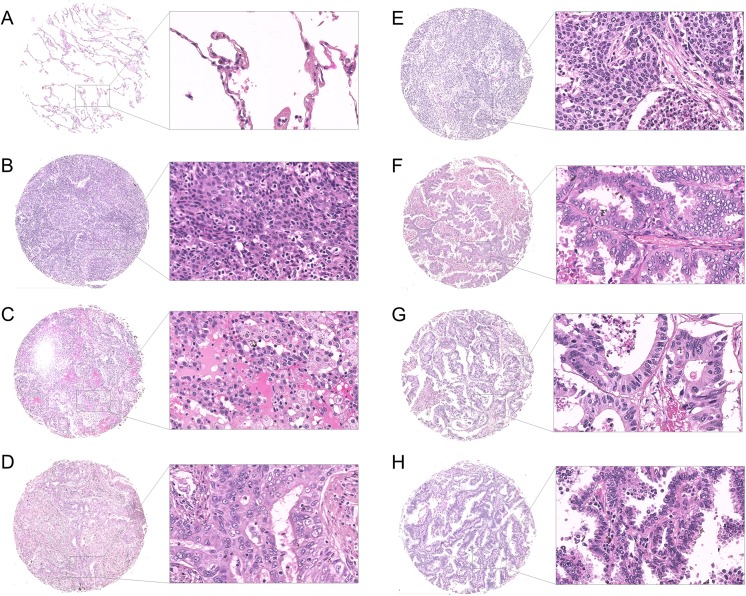
Morphological analysis of human lung specimens. Representative images of human lung sections stained with hematoxylin and eosin based on their pathology. (A) Healthy donor, (B) squamous cell carcinoma, (C) adenocarcinoma, (D) adenosquamous carcinoma, (E) small cell carcinoma, (F) papillary adenocarcinoma, (G) metastatic adenocarcinoma, and (H) bronchioloalveolar carcinoma. Scale bar = 250 μm.

To characterize lung tumor morphology, human lung tumor microarrays were stained with hematoxylin and eosin. Fig 1 shows representative stained tissue specimens according to their pathology; healthy, squamous cell carcinoma, adenocarcinoma, adenosquamous carcinoma, small cell carcinoma, papillary adenocarcinoma, metastatic adenocarcinoma, and bronchioloalveolar carcinoma. All specimens were subjected to additional histopathological analysis.

### Analysis of the Tumor Microenvironment in Human Lung Cancer Tissues

#### T lymphocytes

**Fig 2:**
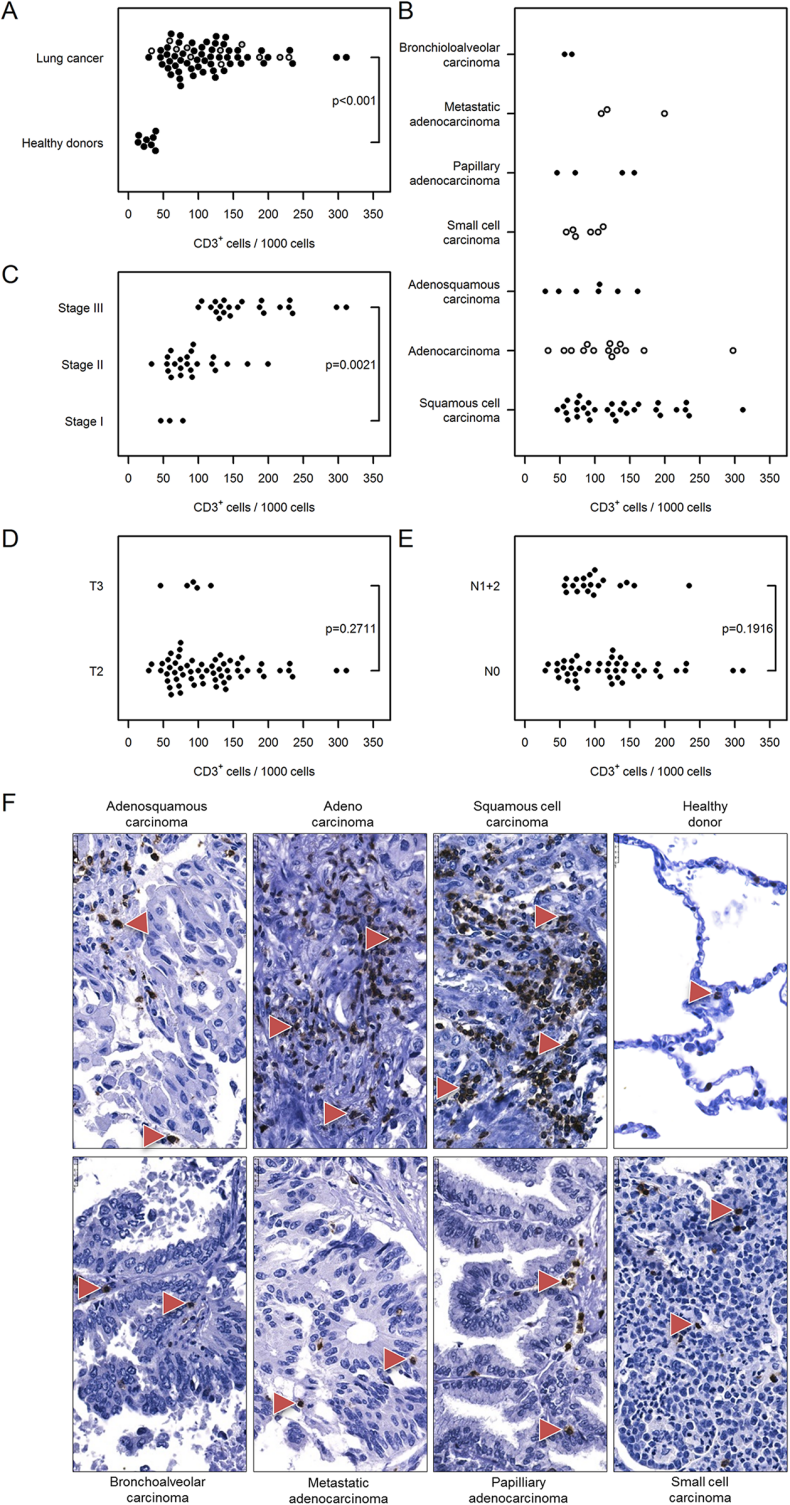
Immunohistochemical analysis and quantification of CD3-positive T lymphocytes in human lung cancer. Human lung cancer tissue array was stained with CD3 antibody to detect T lymphocytes. (A) Quantification of CD3^+^ cells in lung cancer vs. healthy donor specimens. (B–E) Quantification of CD3^+^ cells based on (B) their pathology, (C) cancer stage, (D) tumor size, and (E) nodal status. Cell numbers are given as CD3-positive cells per 1000 cells. (F) Representative images of human lung sections stained with CD3 antibody based on their pathology. Scale bar = 25 μm.

Infiltration of T lymphocytes into human lung tissue was assessed by immunohistochemical analysis using the CD3 antibody. We observed an increased number of CD3^+^ T cells in lung cancer [mean, 118 cells/per 1000 cells; 95% CI, 105–133; here after mentioned as 118 (105…133)] compared with healthy donor lungs [28 (14…57); Fig 2A]. The infiltration of CD3^+^ T lymphocytes was independent of cancer type (Fig 2B and 2F), but the number of infiltrating cells was higher in later stages of lung cancer [173 (151…199) stage III vs. 61(33…114) stage I lung cancer, Fig 2C]. The number of CD3^+^ T cells in tumor samples was independent of tumor size [T2: 119 (104…136] vs. T3: 88 (52…149)] and nodal status [N0: 123 (106…143) vs. N1+2: 101 (79…130), Fig 2D and 2E] based on TNM staging.

**Fig 3:**
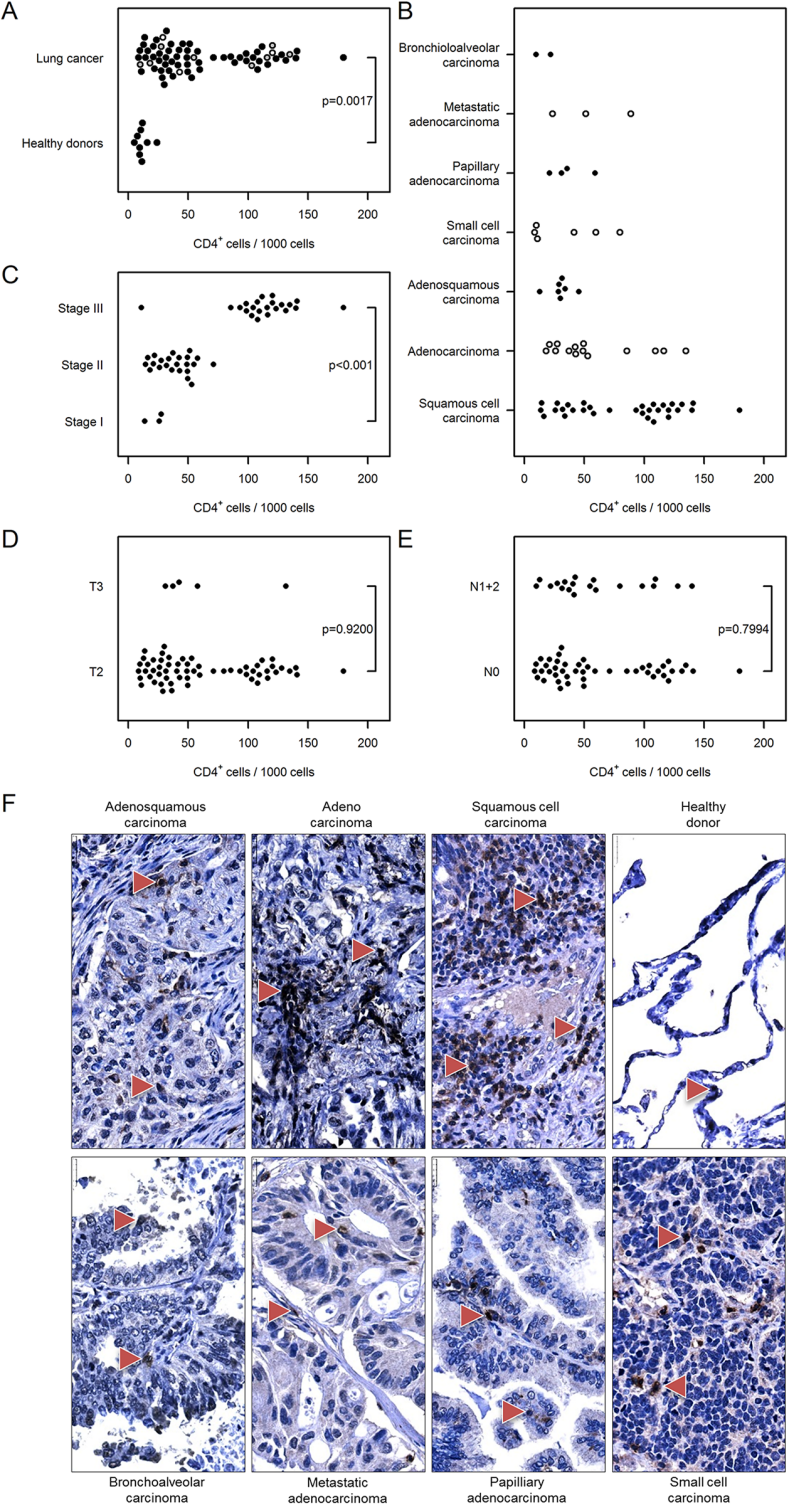
Immunohistochemical analysis and quantification of CD4-positive T lymphocytes in human lung cancer. Human lung cancer tissue array was stained with CD4 antibody to detect T helper cells. (A) Quantification of CD4^+^ cells in lung cancer vs. healthy donor specimens. (B–E) Quantification of CD4^+^ cells based on (B) their pathology, (C) cancer stage, (D) tumor size, and (E) nodal status. Cell numbers are given as CD4-positive cells per 1000 cells. (F) Representative images of human lung sections stained with CD4 antibody based on their pathology. Scale bar = 25 μm.

**Fig 4:**
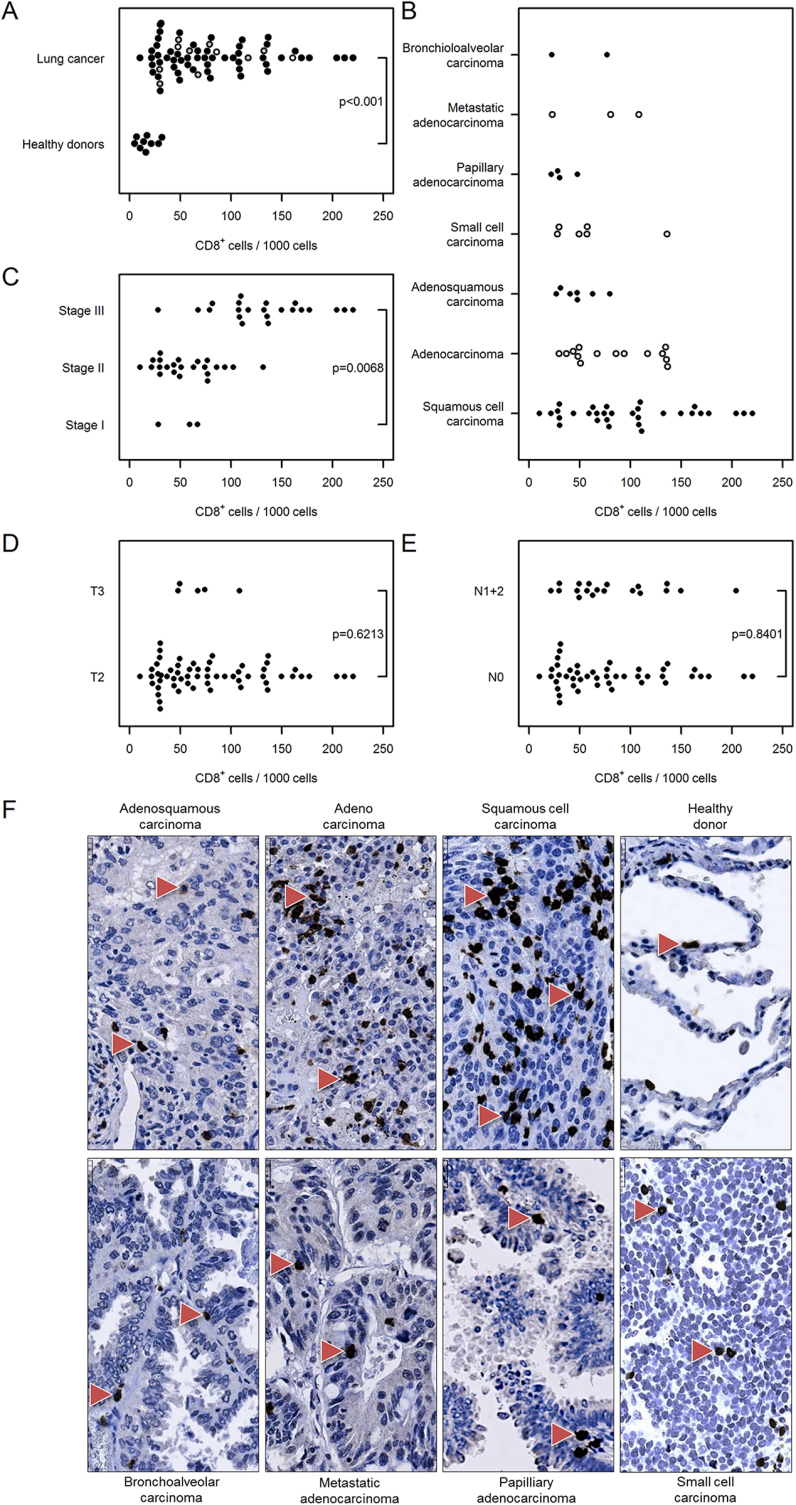
Immunohistochemical analysis and quantification of CD8-positive T lymphocytes in human lung cancer. Human lung cancer tissue array was stained with CD8 antibody to detect cytotoxic T lymphocytes. (A) Quantification of CD8^+^ cells in lung cancer vs. healthy donor specimens. (B–E) Quantification of CD8^+^ cells based on (B) their pathology, (C) cancer stage, (D) tumor size, and (E) nodal status. Cell numbers are given as CD8-positive cells per 1000 cells. (F) Representative images of human lung sections stained with CD8 antibody based on their pathology. Scale bar = 25 μm.

To further evaluate the distribution of T lymphocyte subpopulations, sections were analyzed by immunostaining for the prevalence of T helper (CD4^+^) and cytotoxic (CD8^+^) T cells. As shown in Fig 3A, the number of CD4^+^ cells was significantly increased in tumor tissue compared with healthy donor tissue [62 (52…72) vs. 12 (4…32)]. The prevalence of T helper cells was independent of cancer type (Fig 3B and 3F), however the number of infiltrating cells was higher in stage III cancer compared with stage I [113 (102…125) vs. 22 (12…42), Fig 3C]. The number of CD4^+^ T lymphocytes in tumor samples was independent of tumor size [T2: 62 (52…75] vs. T3: 60 (32…114)] and nodal status [N0: 63 (51…77) vs. N1+2: 60 (43…83), Fig 3D and 3E]. Similar results were obtained for infiltrating cytotoxic T lymphocytes, with a higher number of CD8^+^ cells in tumor tissues compared with healthy controls [80 (69…93) vs. 17 (7…41)] and higher CD8^+^ cell infiltration in stage III vs. stage I lung cancer [132 (114…154) vs. 51 (27…98), Fig 4A and 4C]. There was no correlation between the prevalence of cytotoxic T cells and cancer type, tumor size or nodal status (Fig 4B, 4D and 4E).

#### Macrophages and mast cells

**Fig 5:**
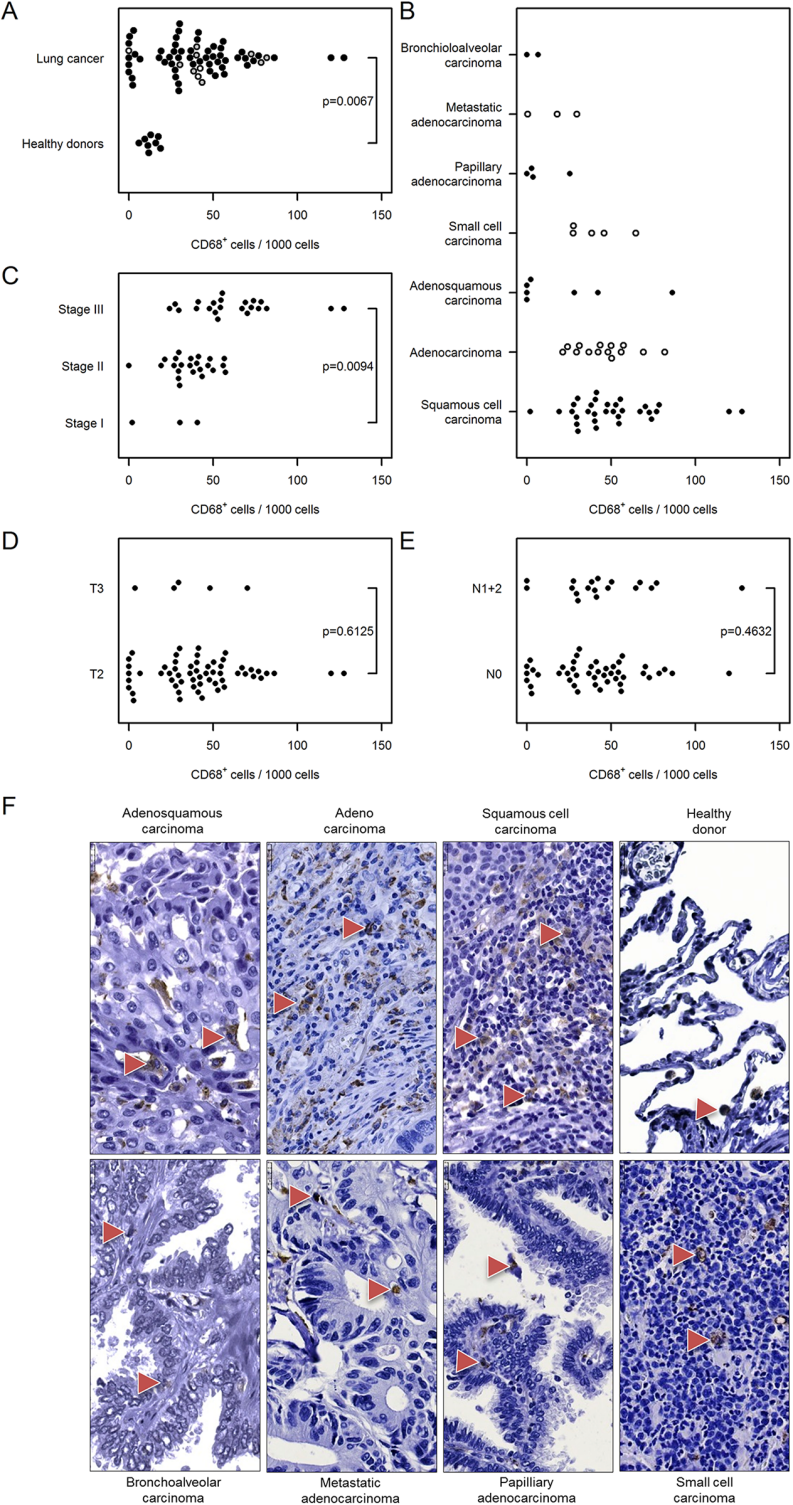
Immunohistochemical analysis and quantification of CD68-positive cells in human lung cancer. Human lung cancer tissue array was stained with CD68 antibody to detect macrophages. (A) Quantification of CD68^+^ cells in lung cancer vs. healthy donor specimens. (B–E) Quantification of CD68^+^ cells based on (B) their pathology, (C) cancer stage, (D) tumor size, and (E) nodal status. Cell numbers are given as CD68-positive cells per 1000 cells. (F) Representative images of human lung sections stained with CD68 antibody based on their pathology. Scale bar = 25 μm.

Tumor-associated macrophages were assessed based on the expression of CD68. We observed an increased number of CD68^+^ cells in lung cancer [39 (30…49)] compared with healthy donor lungs [5 (1…34); Fig 5A]. The number of tumor-associated macrophages also correlated with cancer stage [stage III: 75 (62…92) vs. stage I: 9 (2…41), Fig 5C] and was independent of cancer type, tumor size, and nodal status (Fig 5B and 5D–5F).

**Fig 6:**
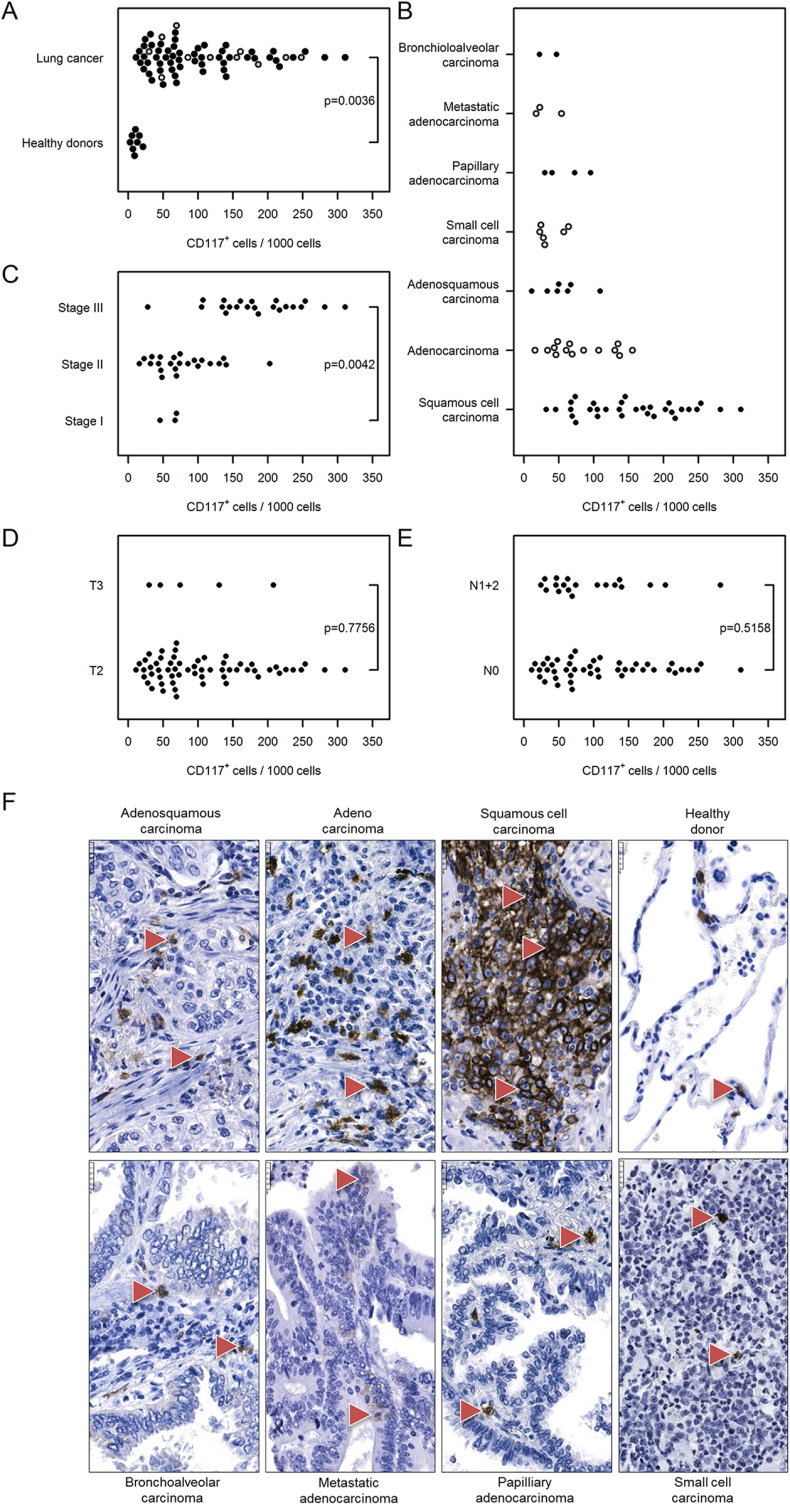
Immunohistochemical analysis and quantification of CD117-positive cells in human lung cancer. Human lung cancer tissue array was stained with CD117 (cKit) antibody to detect mast cells. (A) Quantification of CD117^+^ cells in lung cancer vs. healthy donor specimens. (B–E) Quantification of CD117^+^ cells based on (B) their pathology, (C) cancer stage, (D) tumor size, and (E) nodal status. Cell numbers are given as CD117-positive cells per 1000 cells. (F) Representative images of human lung sections stained with CD117 antibody based on their pathology. Scale bar = 25 μm.

Next, we assessed the number of mast cells in the tumor tissue based on CD117 (cKit) immunodetection. As shown in Fig 6A, the number of mast cells was higher in the tumor tissue compared with healthy donor tissue [103 (88…122) vs. 11 (2…48)] and was substantially elevated in stage III cancer compared with stage I [183 (157…213) vs. 61 (30…124), Fig 6C]. We did not detect any differences among samples according to cancer type, tumor size or nodal status (Fig 6B and 6D–6F).

#### Granulocytes

**Fig 7:**
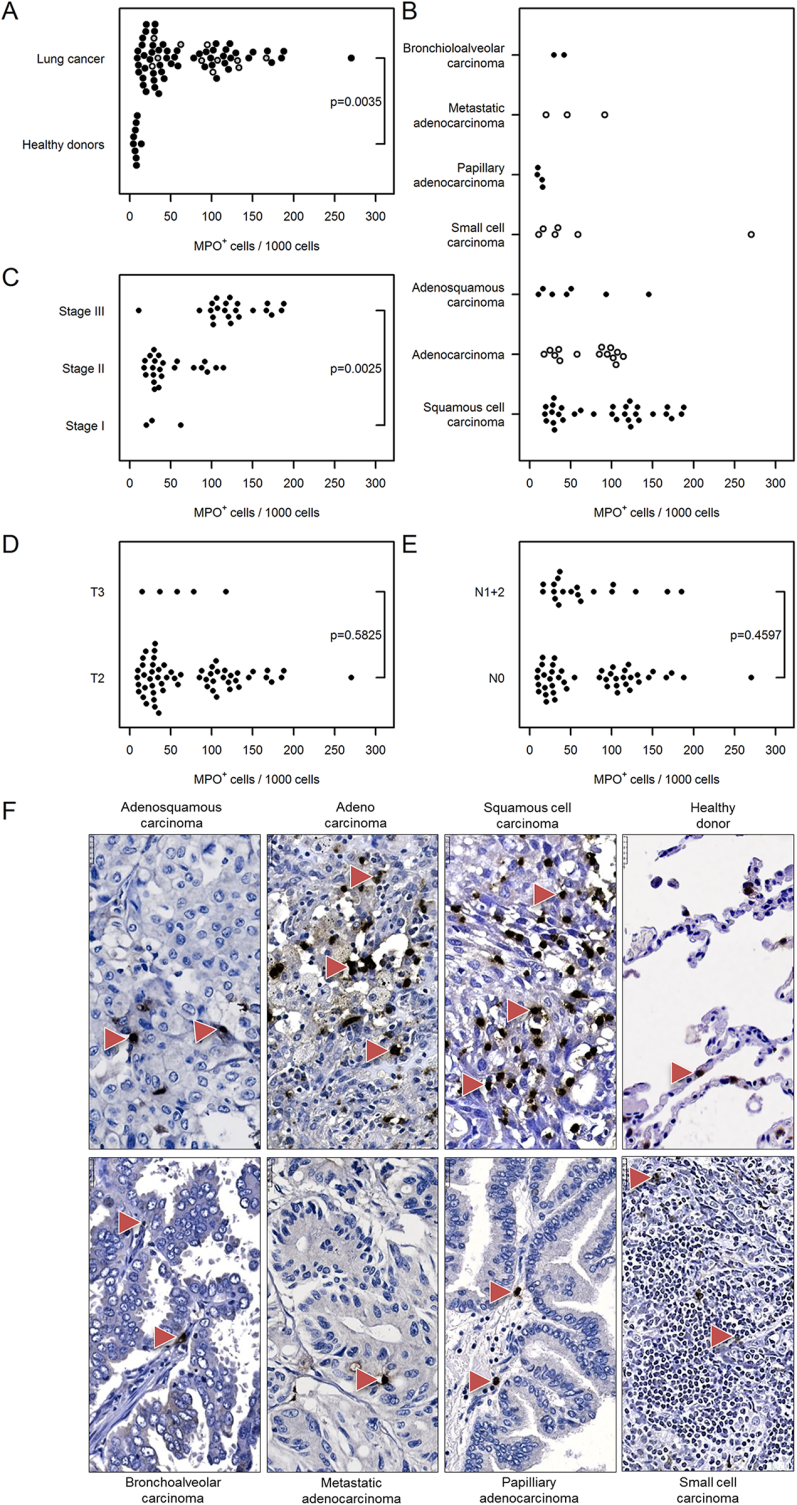
Immunohistochemical analysis and quantification of MPO-positive cells in human lung cancer. Human lung cancer tissue array was stained with MPO antibody to detect neutrophil granulocytes. (A) Quantification of MPO^+^ cells in lung cancer vs. healthy donor specimens. (B–E) Quantification of MPO^+^ cells based on (B) their pathology, (C) cancer stage, (D) tumor size, and (E) nodal status. Cell numbers are given as MPO-positive cells per 1000 cells. (F) Representative images of human lung sections stained with MPO antibody based on their pathology. Scale bar = 25 μm.

The number of infiltrating neutrophil granulocytes was determined based on MPO immunoreactivity. Similar to the previous results, we found elevated numbers of neutrophils in the cancer tissue compared with healthy donor lungs [74 (62…88) vs. 8 (2…34), Fig 7A] and in the later stages of cancer [stage III: 125 (107…145) vs. stage I: 37 (17…78), Fig 7C]. Similarly, there was no correlation between neutrophil number and cancer type, tumor size or nodal status (Fig 7B and 7D–7F).

#### B cells and dendritic cells

**Fig 8:**
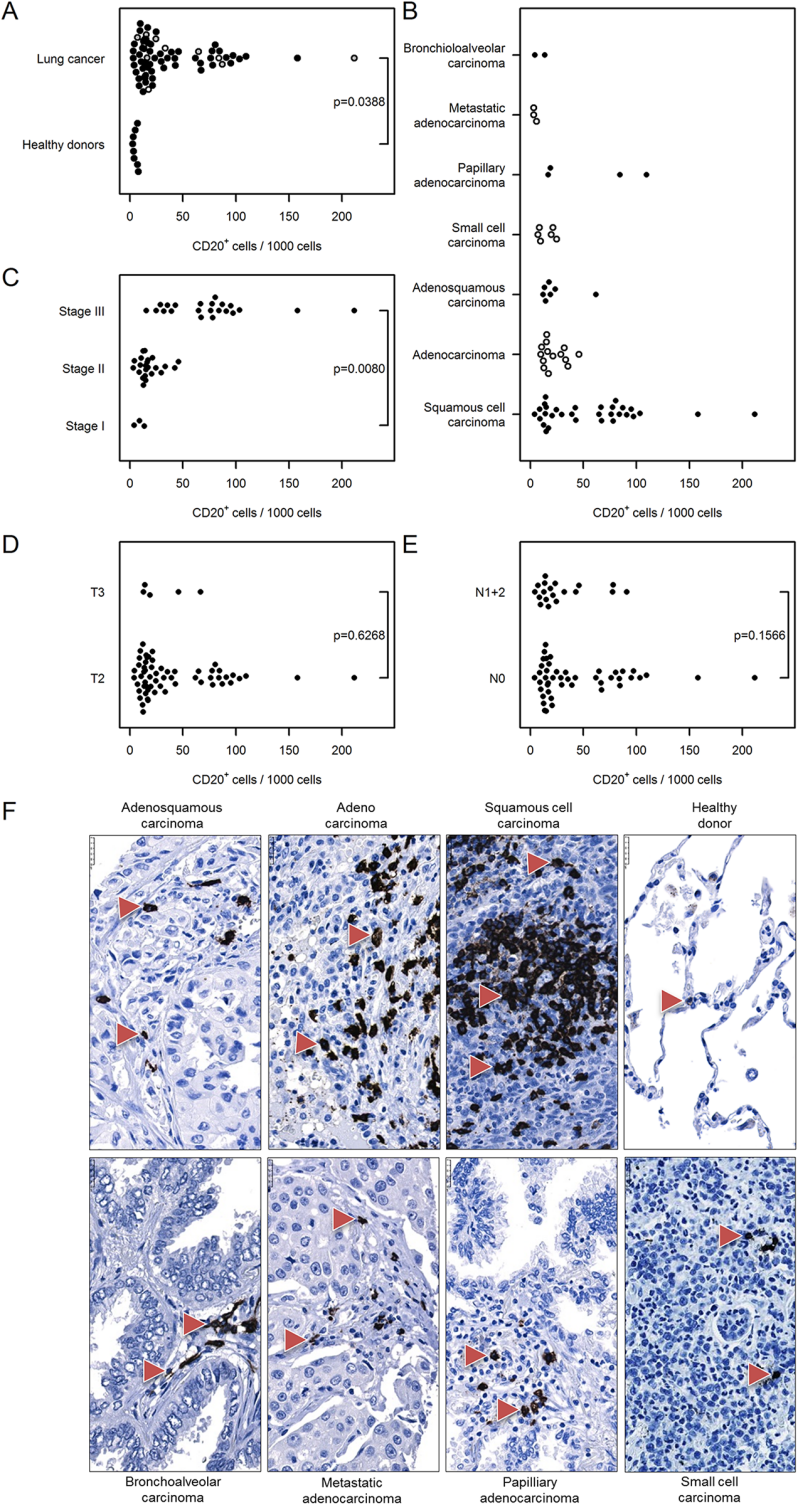
Immunohistochemical analysis and quantification of CD20-positive cells in human lung cancer. Human lung cancer tissue array was stained with CD20 antibody to detect B cells. (A) Quantification of CD20^+^ cells in lung cancer vs. healthy donor specimens. (B–E) Quantification of CD20^+^ cells based on (B) their pathology, (C) cancer stage, (D) tumor size, and (E) nodal status. Cell numbers are given as CD20-positive cells per 1000 cells. (F) Representative images of human lung sections stained with CD20 antibody based on their pathology. Scale bar = 25 μm.

**Fig 9:**
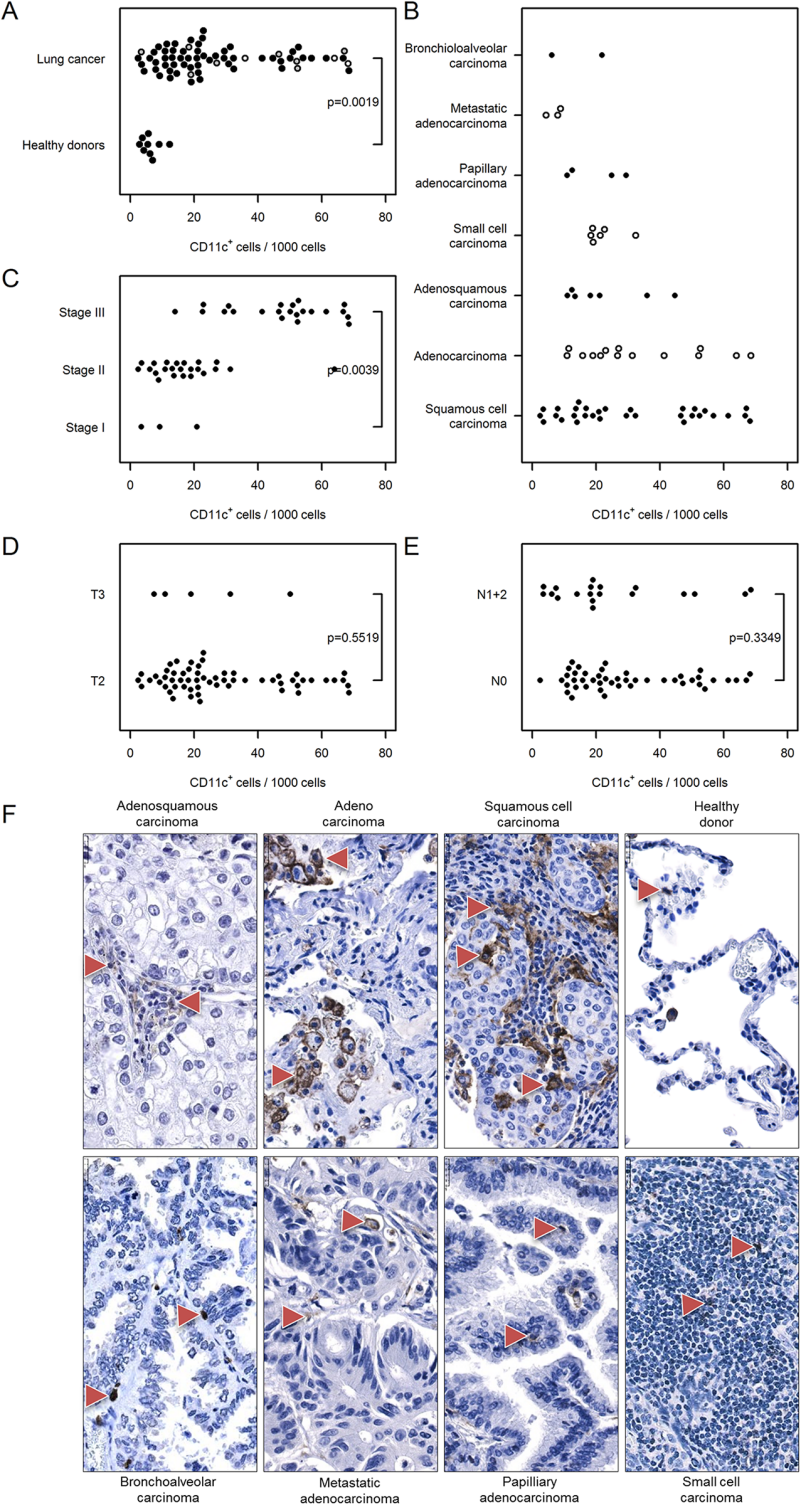
Immunohistochemical analysis and quantification of CD11c-positive cells in human lung cancer. Human lung cancer tissue array was stained with CD11c antibody to detect dendritic cells. (A) Quantification of CD11c^+^ cells in lung cancer vs. healthy donor specimens. (B–E) Quantification of CD11c^+^ cells based on (B) their pathology, (C) cancer stage, (D) tumor size, and (E) nodal status. Cell numbers are given as CD11c-positive cells per 1000 cells. (F) Representative images of human lung sections stained with CD11c antibody based on their pathology. Scale bar = 25 μm.

Next, based on the expression of CD20 and CD11c, we assessed the number of tumor-infiltrating B cells and of dendritic cells (along with monocytes, macrophages, and neutrophils) respectively. We found an increase in CD20^+^ B cells within the tumor tissue compared with the healthy specimens [39 (30…49) vs. 5 (1…34), Fig 8A]. The number of CD20^+^ cells was also elevated in stage III vs. stage I cancer samples [75 (62…92) vs. 9 (2…41), Fig 8C]. The numbers of of CD20^+^ B cells in cancer tissue was independent of cancer type and tumor size (Fig 8B, 8D and 8F). A considerably higher number of infiltrating B cells was detected in N0 tumor samples compared with N1+2 samples [45 (34…59) vs. 29 (18…49] (Fig 8E). Likewise, the number of dendritic cells was substantially elevated within the tumor tissue as assessed by CD11c immunostaining [28 (24…32) vs. 6 (3…16), Fig 9A]. Further, the number of CD11c^+^ cells in the tumor tissue was independent of cancer type (Fig 9B and 9F), although the number of infiltrating cells was elevated in later stages of lung cancer [47 (40…56) stage III vs. 11(4…28) stage I lung cancer, Fig 9C]. The number of CD11c^+^ dendritic cells in tumor samples was independent of tumor size and nodal status (Fig 9D and 9E).

#### MUM1– and PD1–positive cells

**Fig 10:**
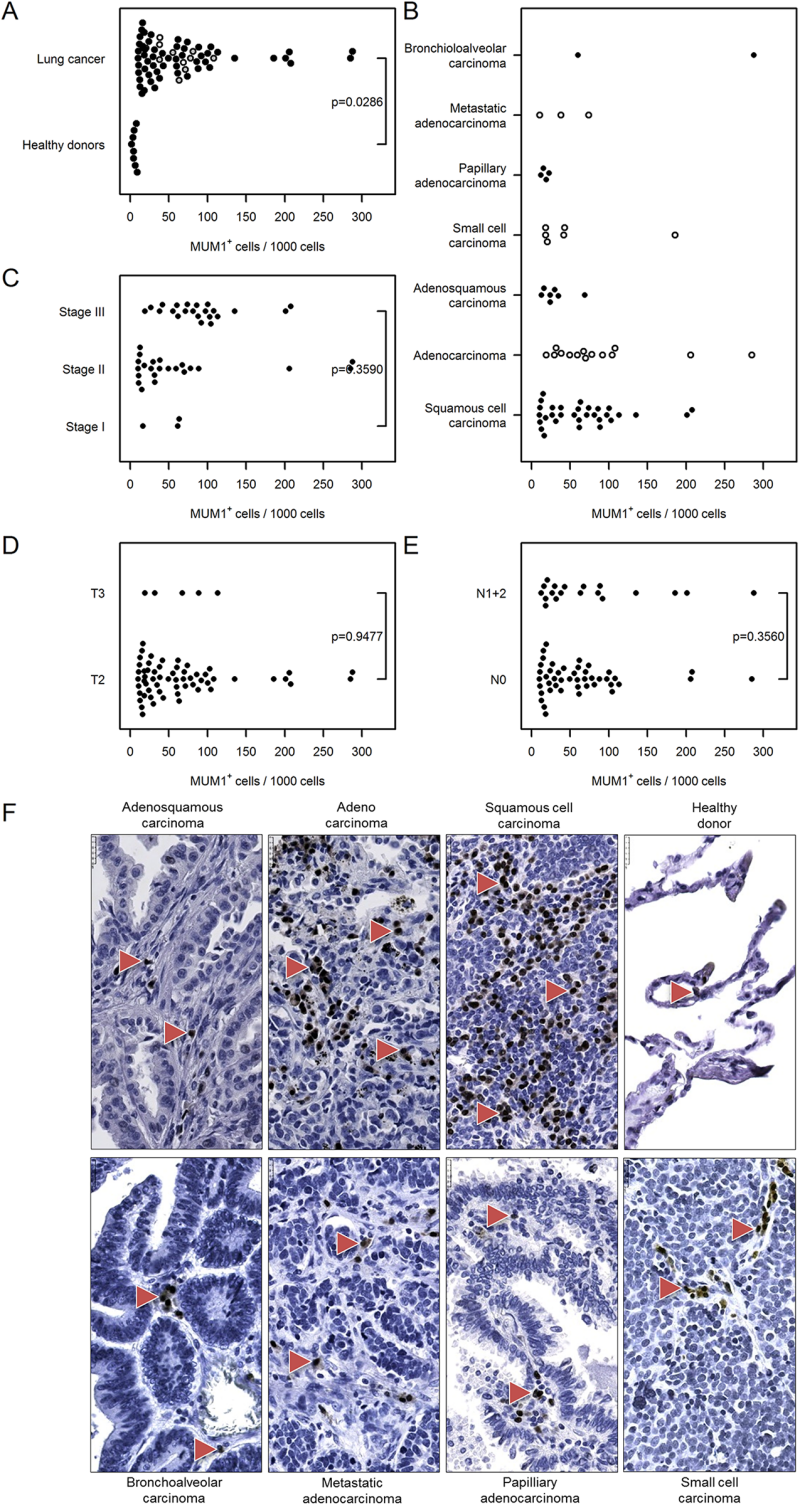
Immunohistochemical analysis and quantification of MUM1–positive cells in human lung cancer. Human lung cancer tissue array was stained with MUM1 antibody to detect plasma cells and activated T cells. (A) Quantification of MUM1^+^ cells in lung cancer vs. healthy donor specimens. (B–E) Quantification of MUM1^+^ cells based on (B) their pathology, (C) cancer stage, (D) tumor size, and (E) nodal status. Cell numbers are given as MUM1–positive cells per 1000 cells. (F) Representative images of human lung sections stained with MUM1 antibody based on their pathology. Scale bar = 25 μm.

**Fig 11:**
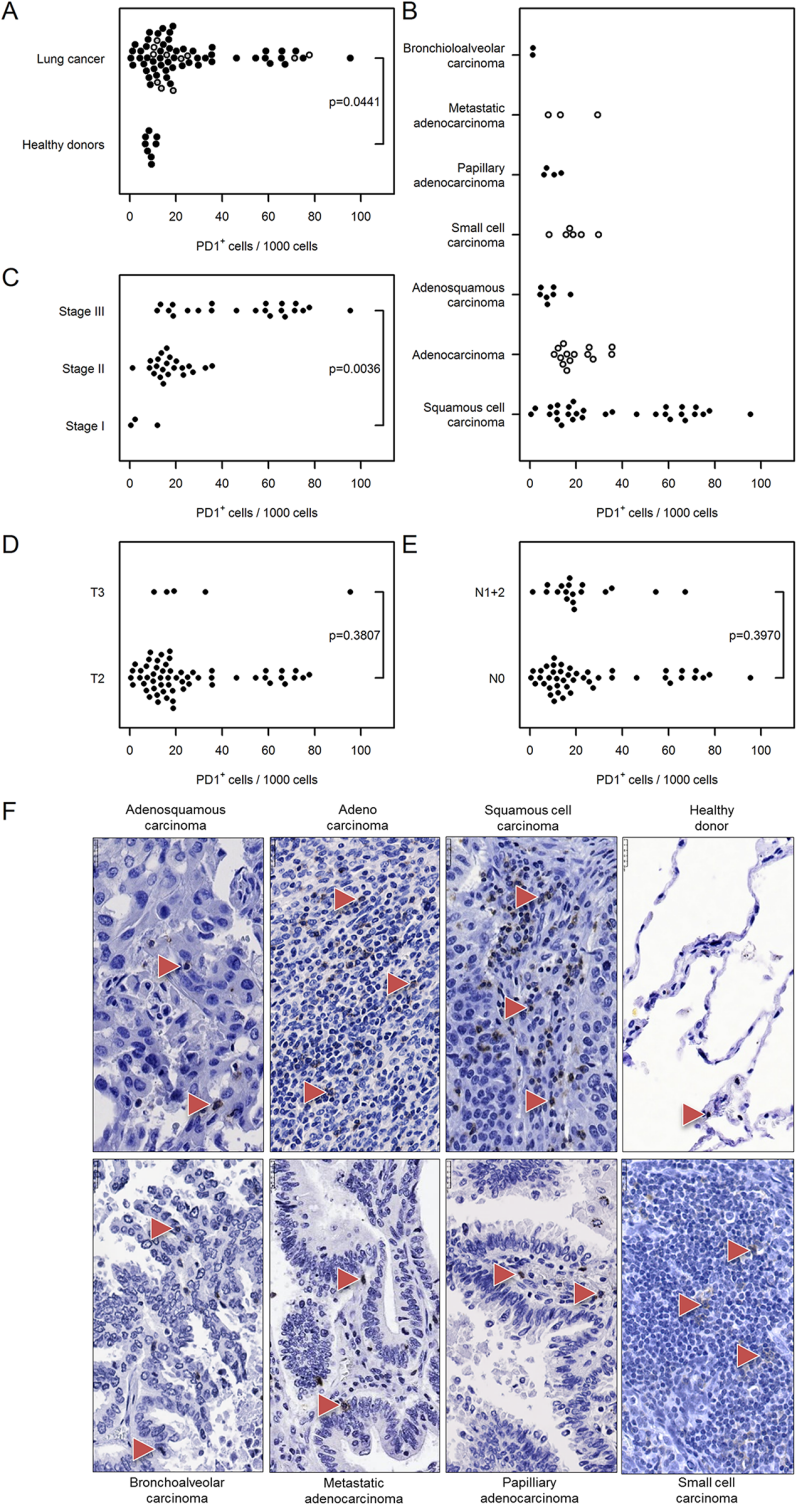
Immunohistochemical analysis and quantification of PD1–positive cells in human lung cancer. Human lung cancer tissue array was stained with PD1 antibody to detect activated T cells, B cells, myeloid cells, and a subset of thymocytes. (A) Quantification of PD1^+^ cells in lung cancer vs. healthy donor specimens. (B–E) Quantification of PD1^+^ cells based on (B) their pathology, (C) cancer stage, (D) tumor size, and (E) nodal status. Cell numbers are given as PD1–positive cells per 1000 cells. (F) Representative images of human lung sections stained with PD1 antibody based on their pathology. Scale bar = 25 μm.

We evaluated the expression levels of two additional immune markers. MUM1 labels plasma cells and activated T cells while PD1 labels activated T cells, B cells, myeloid cells and a subset of thymocytes. Both MUM1–positive cells and PD1–positive cells were elevated in cancer tissue compared with control lungs [MUM1^+^ cells: 65 (52…81) vs. 6 (1…50), PD1^+^ cells: 26 (21…32) vs. 9 (3…24), Figs 10A and 11A]. The prevalence of MUM1–positive cells was independent of the cancer type, stage, tumor size and nodal status (Fig 10B–10F). The number of PD1^+^ cells correlated with the cancer stage [stage III: 49 (41…58) vs. stage I: 5 (1…22), Fig 11C], but there was no correlation with regard to cancer type, tumor size or nodal status (Fig 11B and 11D–11F).

## Discussion

In this study, employing tissue arrays and immunohistochemistry, we comprehensively analyzed stromal cell composition in different human lung cancer types, grade and stage. In combination with an immunohistochemical analysis, we also employed a cyto-/histomorphological assessment of cells. This combination allowed us to classify/subclassify tumors accurately and to perform a high throughput analysis of stromal cell composition in tumor types with inter-individual variability. Importantly, we observed extensive immune and inflammatory cell infiltration in human lung cancer samples. We comprehensively characterized and quantified T lymphocytes (CD3+), cytotoxic-T cells (CD8+), T-helper cells (CD4+), B cells (CD20+), macrophages (CD68+), mast cells (CD117+), mononuclear cells (CD11c+), plasma cells and activated-T cells (MUM1+), activated-T cells, B cells, and myeloid cells (PD1+), and neutrophilic granulocytes (myeloperoxidase+) in different lung cancer types, cancer stages, and nodal status.

In agreement with the studies of Ruffini *et al*. [16], we found an increase in the number of CD3^+^ lymphocytes in lung cancer tissue compared with healthy donor lungs. However, analysis of how T cells affect clinical outcome has often yielded contradictory results. Kilic *et al*. [18] reported that higher levels of tumor-infiltrating lymphocytes within large lung tumors correlate with a decreased risk of disease recurrence, whereas Kawai *et al*. reported no correlation between the number of tumor-infiltrating lymphocytes and patient survival [17]. In our study, in addition to general analysis of tumor-infiltrating lymphocytes, analysis of specific T-cell subsets demonstrated a significant increase in tumor-infiltrating T helper cells (CD4^+^) as well as cytotoxic T cells (CD8^+^) compared with healthy donor lungs. Tumor-infiltrating lymphocytes are thought to play an important role in anticancer immunosurveillance [17]. CD8^+^ T cells recognize and destroy cancer cells while CD4^+^ cells aid CD8^+^ T cells in tumor rejection. Therefore, their number and localization in tumor tissue may influence tumorigenicity [26]. Although high in number, CD8^+^ T cells that infiltrate lung tumors may be dysfunctional due to tumor microenvironmental factors which may subsequently lead to reduced numbers of effector CD8^+^ T cells [27]. These altered CD8^+^ T cells may even release compounds that promote tumor progression. Thus, a deeper understanding and dissection of the contribution of different CD4^+^ and CD8^+^ T cell subpopulations (e.g., Th1, Th2, Th17, Tc9, Tc17) is necessary.

Among phagocytes and granulocytes, we observed that higher numbers of infiltrating macrophages, mast cells, and neutrophils correlated positively with tumor stage in human lung cancer patients. The density of macrophages within tumor islets and the ratio of macrophages situated in these islets to the stromal macrophages are positive prognostic factors for patient survival [17, 28]. In contrast, other studies have suggested that both M1 and M2 macrophages favor carcinogenesis [29] and their numbers within the cancer may be negative prognostic factors [30]. We observed that macrophage infiltration correlated positively with tumor stage and nodal status/ metastasis in human lung cancer patients suggesting an important contribution of these tumor-associated macrophages in lung cancer progression and metastasis. To confirm the importance of macrophages, future studies should address the subtypes of macrophages present in the microenvironment of the lung tumor since recent studies suggest that during carcinogenesis, macrophages may polarize to M1 (anti-tumorigenic) and M2 (contributing to carcinogenesis) subtypes and thus can exert differential effects [31]. Furthermore, we need to understand how the bidirectional cross talk between macrophages and cancer cells influences these cells as well as dissecting the underlying molecular mechanisms [32]. We recently provided evidence that macrophage and cancer cell cross talk via CCR2 and CX3CR1, a fundamental mechanism driving lung cancer [12]. These findings suggest that the therapeutic strategy of blocking CCR2 and CX3CR1 may prove beneficial for halting lung cancer progression.

Regarding mast cells, Welsh and colleagues [28] showed that an increased islet/stromal mast cell ratio is an advantageous independent prognostic factor, whereas Kawai *et al*. [17] found no correlation with clinical outcome. Here, we showed that the mast cell number was higher in tumor tissue compared with healthy donors and was substantially elevated in stage III cancer compared with stage I.

Neutrophil granulocytes have received increased attention as a new type of tumor-infiltrating immune cell that plays a role in tumor growth. However, little is known about their role in lung cancer. It has been suggested that increased numbers of neutrophils have been observed in the bronchoalveolar lavage fluid of patients with bronchioloalveolar carcinoma, and serves as an independent predictor of clinical outcome [33]. Here we show an elevated number of neutrophils in lung cancer specimens compared with healthy lung and a strong association with advanced lung cancer stages.

Dendritic cells are a potent, heterogeneous group of antigen-presenting cells that are important for primary immune responses to carcinoma. We found a significant increase in CD11c-positive cells within the tumor mass in advanced-stage cancer samples compared with healthy donors. We speculate that the vast majority of these cells correspond to dendritic cells, but we cannot exclude the possibility that a portion may be monocytes, macrophages, or neutrophils, which also express CD11c [34]. Although we did not assess the maturity of dendritic cells, previous work [35] hinted that at least some of the tumor-infiltrating dendritic cells display an immature phenotype in non–small-cell lung cancer and that this may be due to several tumor-derived factors.

Among the other antigen-presenting cells, we found an increased number of CD20-positive B cells in lung cancer samples compared with healthy donor tissue. As the role of B cells in cancer progression is rather debatable [16, 19, 36], we additionally assessed the number of MUM1–positive plasma cells in tumor and healthy human lung tissue. Plasma cells originate from B cells upon their encounter with a foreign antigen and are the sole producers of antibodies [37]. We found increased numbers of plasma cells in cancer tissue compared with donor lungs, a result that was independent of the cancer stage as well as other measured parameters. The role of plasma cells in solid tumors has not been intensively investigated, and thus only a few reports of their role in lung cancer exist. Lohr and colleagues showed that infiltration of mature plasma cells into tumor tissue is associated with prolonged survival [38]. Another study reported infiltration of IgG4-positive plasma cells in specimens of stage I squamous cell carcinoma that were associated with favorable prognosis [39].

PD1 is an immunoglobulin superfamily member found primarily on immature CD4^−^CD8^−^ thymocytes during T-cell receptor-β rearrangement. Upon activation, PD1 may also be expressed on peripheral T helper and cytotoxic T cells, B lymphocytes, natural killer T cells, and monocytes [40]. PD1 function is best characterized in T cells, where it plays a vital role in the induction and maintenance of anergy and peripheral tolerance of T cells by inhibiting their proliferation and cytokine production [40, 41]. Here, we show a significantly increased number of PD1–positive cells in lung cancer tissue compared with healthy control lungs. The number of PD1^+^ cells correlated with cancer stage but was independent of cancer type, tumor size and nodal status. Our finding that the number of PD1^+^ cells correlated with the cancer stage is of interest, as recent studies suggest that PD1 signaling status (e.g. PD-ligand 1 expression) may be a potential predictive biomarker for anti-PD-1 therapy [42]. In conclusion, the immune infiltrates are of major importance in development and progression of lung cancer and in determining prognosis of patients with lung cancer.

### Strengths and limitations of the study

In this study, employing tissue arrays and immunohistochemistry, we comprehensively analyzed stromal cell composition in different human lung cancer types, grade and stage. As demonstrated in our study we were successful in high throughput analysis of stromal cell composition in tumor types with inter-individual variability using tissue arrays. Therefore, application of a tissue array with lung carcinoma cores is suitable and feasible. However, with this technology, our ability to characterize in detail the tumor heterogeneity is limited. As we have only duplicate cores per sample in the tissue arrays used, the issue of tumor heterogeneity cannot be addressed in detail. Moreover, it must be taken into account that in a few instances, cores do not contain tumor, thus a careful morphological checkup is necessary. In addition, we can detect unspecific staining by suboptimal immunohistochemical labeling, which can be circumvented by correlation to cell morphology and background. Thus we believe this technology requires well-experienced personnel. One other alternative method that could be developed is multiparameter assessment of immune and inflammatory cell composition of human lung cancer stroma using flow cytometry or confocal microscopy, which is beyond the scope of the present study.
